# Comparison of the Proportions of Female and Male Corresponding Authors in Preprint Research Repositories Before and During the COVID-19 Pandemic

**DOI:** 10.1001/jamanetworkopen.2020.20335

**Published:** 2020-09-17

**Authors:** Mackenzie R. Wehner, Yao Li, Kevin T. Nead

**Affiliations:** 1 Department of Health Services Research, University of Texas MD Anderson Cancer Center; 2 Department of Dermatology, University of Texas MD Anderson Cancer Center, Houston, Texas; 3 Department of Epidemiology, University of Texas MD Anderson Cancer Center, Houston, Texas; 4 Department of Radiation Oncology, University of Texas MD Anderson Cancer Center, Houston, Texas

## Abstract

This cross-sectional study assesses whether there were differences in the proportions of male and female corresponding authors in bioRxiv and medRxiv associated with the COVID-19 pandemic.

## Introduction

There is substantial interest regarding the effects of the coronavirus disease 2019 (COVID-19) pandemic on academic productivity. Many researchers are working from home, and schools and childcare centers are closed, leading to childcare disruptions. Research has demonstrated that women in academia perform a greater proportion of domestic work than men, including in dual academic career partnerships.^1,2^ We hypothesized that remote working and disruptions to family support structures secondary to the pandemic may have been disproportionately associated with the academic productivity of women. We examined changes in the proportion of female corresponding authors in bioRxiv (biorxiv.org) and medRxiv (medrxiv.org), which are online archive and distribution services for unpublished preprint research in the life and health sciences, respectively, before vs during the COVID-19 pandemic.^3^

## Methods

We conducted a cross-sectional study using web-scraped metadata from medRxiv from June 6, 2019 (inception), to May 5, 2020, and bioRxiv from January 1, 2019, to May 20, 2020. This study was determined to be exempt from institutional review board approval by MD Anderson Cancer Center. This study followed the Strengthening the Reporting of Observational Studies in Epidemiology (STROBE) reporting guideline.

We determined corresponding authors’ gender for 49 924 of 51 249 articles (97%) using first name via Gender API, which includes data on more than 2 million names across 177 countries and has demonstrated superior performance vs similar gender inference services.^4^ We determined corresponding authors’ institution country of origin using a combination of listed country, the Nature Index, and manual entry. We analyzed the most recent version of each article and included data from months with at least 100 unique submissions.

We conducted a test for trend using the Somers Delta (Somers D; *somersd*) in Stata, version 16.1 (StataCorp) to assess whether the absolute difference in the percentages of publications between male and female corresponding authors (ie, *gender gap*) changed over time. Tests were considered to be significant with a 2-sided *P* < .05.

## Results

**Figure 1.**
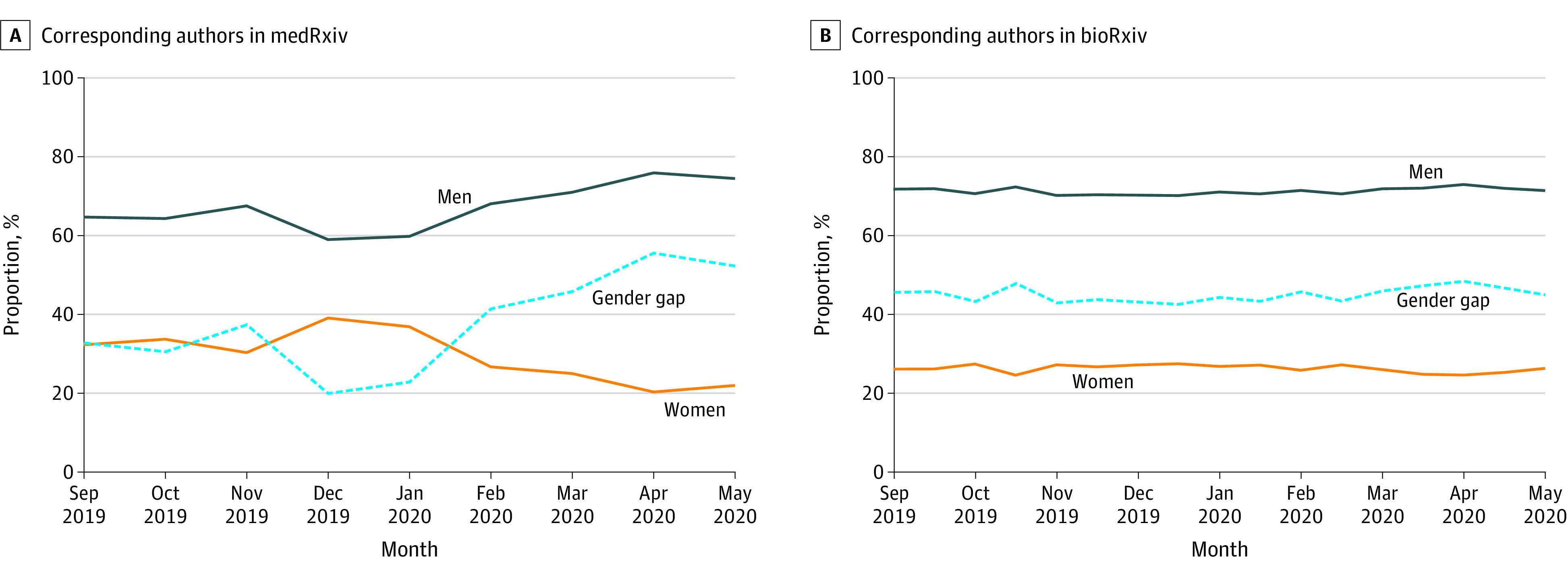
Gender of Corresponding Authors in medRxiv and bioRxiv Over Time The absolute difference of the percentages of male and female corresponding authors are presented as the *gender gap*. Percentages do not always add to 100% because the gender of some corresponding authors was unknown. The time scale for medRxiv is shorter than that of bioRxiv owing to the more recent inception of medRxiv.

**Figure 2.**
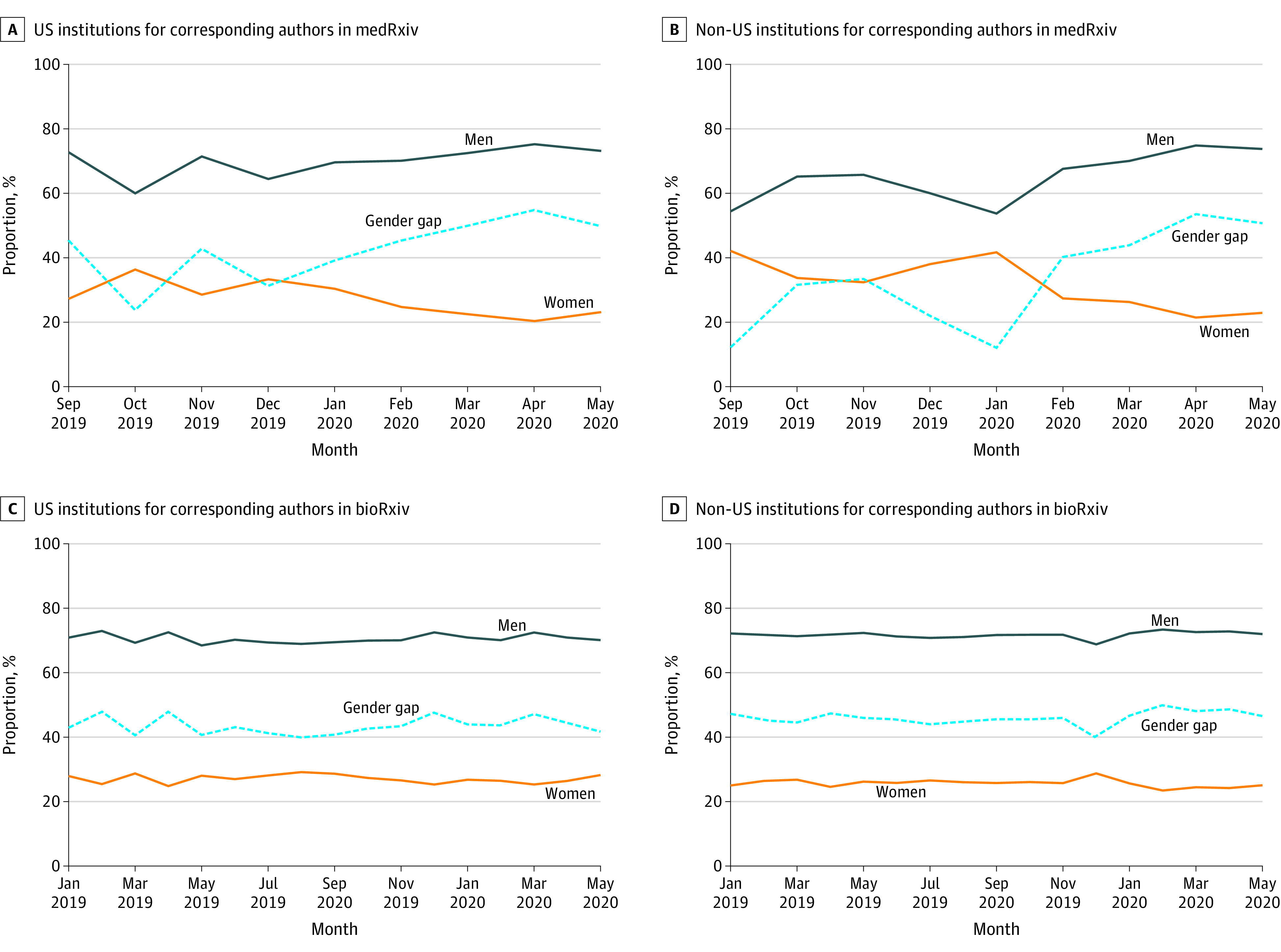
Gender of Corresponding Authors in medRxiv and bioRxiv Over Time Stratified by Institution Country The absolute difference of the percentages of male and female corresponding authors are presented as the *gender gap*.

The percentages of male and female corresponding authors and the gender gap in bioRxiv (46 101 articles) and medRxiv (5148 articles) over time are presented in Figure 1. We observed a statistically significant increase over time in the gender gap in medRxiv (Somers D, 0.14; 95% CI, 0.03 to 0.24) but not in bioRxiv (Somers D, 0.06; 95% CI, −0.01 to 0.12). During the pandemic, the gender gap in medRxiv increased from 23% in January 2020 to 55% in April 2020 and in bioRxiv changed from 46% in January 2020 to 47% in April 2020. We identified corresponding authors’ institution country for 89% of articles, and the numbers of US- and non-US–based analyses were consistent (medRxiv US: n = 1377; Somers D, 0.04 [95% CI, –0.07 to 0.15]; medRxiv non-US: n = 3804; Somers D, 0.17 [95% CI, 0.01 to 0.23]; bioRxiv US: n = 17 996; Somers D, 0.01 [95% CI, –0.05 to 0.07]; bioRxiv non-US: n = 23 209; Somers D, 0.04 [95% CI, –0.02 to 0.10]) (Figure 2).

## Discussion

The present analysis focused on corresponding authors to investigate the consequences of the COVID-19 pandemic for established investigators because this group may encounter unique challenges requiring specialized solutions (eg, tenure-clock extensions).

We observed a statistically significant increase in the corresponding author gender gap in medRxiv (23% in January 2020 to 55% in April 2020), which was unlikely to reflect seasonal variation alone. We did not observe a statistically significant change in the corresponding author gender gap in bioRxiv (46% in January 2020 to 47% in April 2020). The results were consistent in US- and non-US–based analyses. The difference in our findings between bioRxiv and medRxiv may reflect distinct populations of researchers contributing to each service.

Limitations of this study include the inference of gender based on first name and that findings among individuals who submit to preprint services may not be generalizable. Further research is needed to determine whether trends in preprint services translate to concrete metrics of academic achievement.

As the COVID-19 pandemic continues to progress, preprint archiving services may be a useful metric to evaluate real-time academic trends. If a clear gender gap develops, as it appears to be doing in health sciences research, academic institutions and funding agencies may need to address the COVID-19 pandemic–related gender disparities.
